# Supplementary material for: Component Parts of Bacteriophage Virions Accurately Defined by a Machine-Learning Approach Built on Evolutionary Features
Source: mSystems. 2021 May 27;6(3):e00242-21. doi: 10.1128/mSystems.00242-21 (PMC8269216; doi:10.1128/mSystems.00242-21)
Supplement: FIG S1 [file msystems.00242-21-sf001.pdf]

**a**

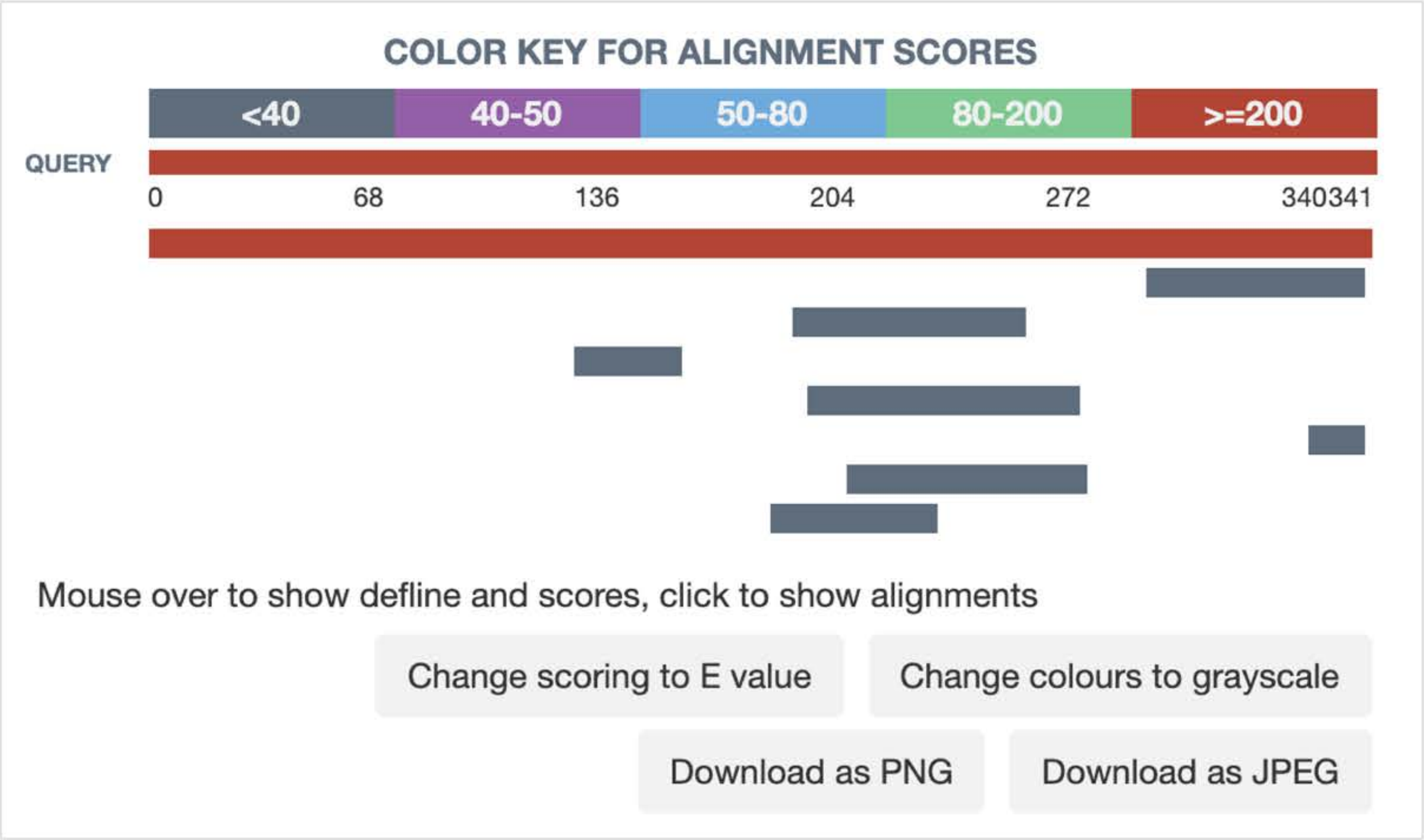


| Description | Max score | Total score | Query cover | E value | Identities |
|---|---|---|---|---|---|
| P68650 Major capsid protein OS=Enterobacteria phage P21 OX=10711 GN=E PE=3 SV=1 | 431 | 431.0 | 99% | 3e-154 | 62% |
| Q01076 Internal virion protein gp20 OS=Salmonella phage P22 OX=10754 GN=20 PE=1 SV=2 | 26.2 | 26.2 | 18% | 0.39 | 27% |
| P10928 Baseplate wedge protein gp10 OS=Enterobacteria phage T4 OX=10665 GN=10 PE=1 SV=1 | 25.4 | 25.4 | 19% | 0.77 | 30% |
| P16009 Baseplate central spike complex protein gp5 OS=Enterobacteria phage T4 OX=10665 GN=5 PE=1 SV=2 | 22.7 | 22.7 | 9% | 4.8 | 29% |
| Q4Z9F2 Tail sheath protein OS=Staphylococcus phage Twort (strain DSM 17442) OX=1283338 PE=3 SV=1 | 22.7 | 22.7 | 22% | 5.3 | 30% |
| P13390 L-shaped tail fiber protein pb1 OS=Escherichia phage T5 OX=10726 GN=ltf PE=1 SV=3 | 22.3 | 22.3 | 4% | 7.0 | 53% |
| P25129 Attachment protein G3P OS=Pseudomonas phage Pf1 OX=2011081 GN=III PE=3 SV=1 | 21.9 | 21.9 | 19% | 7.8 | 28% |
| Q6QGF0 Probable central straight fiber OS=Escherichia phage T5 OX=10726 GN=D17 PE=2 SV=1 | 21.9 | 21.9 | 13% | 10.0 | 36% |

Download as CSV Download as PNG Download as JPEG

**P68650 Major capsid protein OS=Enterobacteria phage P21 OX=10711 GN=E PE=3 SV=1**

| Score: | Expect: | Identities: | Positives: | Gaps: |
|---|---|---|---|---|
| 431 | 3e-154 | 62% | 73% | 0% |

```
Query    1  MSMYTTAQLLAANEQKFKFDPLFLRLFFRESYPFTTEKVYLSQIPGLVNMALYVSPIVSGEVIRSRGGSTSEFTPGYV
            M ++TT QLL   EQK KF  LFL LFFR +  F TE+V L +I G   + A YVSP+V G+V+R RGG T    PGYV
Subject  1  MGLFTTRQLLGYTEQKVKFRALFLELFFRRTVNFHTEEVMLDKITGKTPVAAYVSPVVEGKVLRHRGGETRVLRPGYV
```

Download as PNG Download as JPEG

**b**

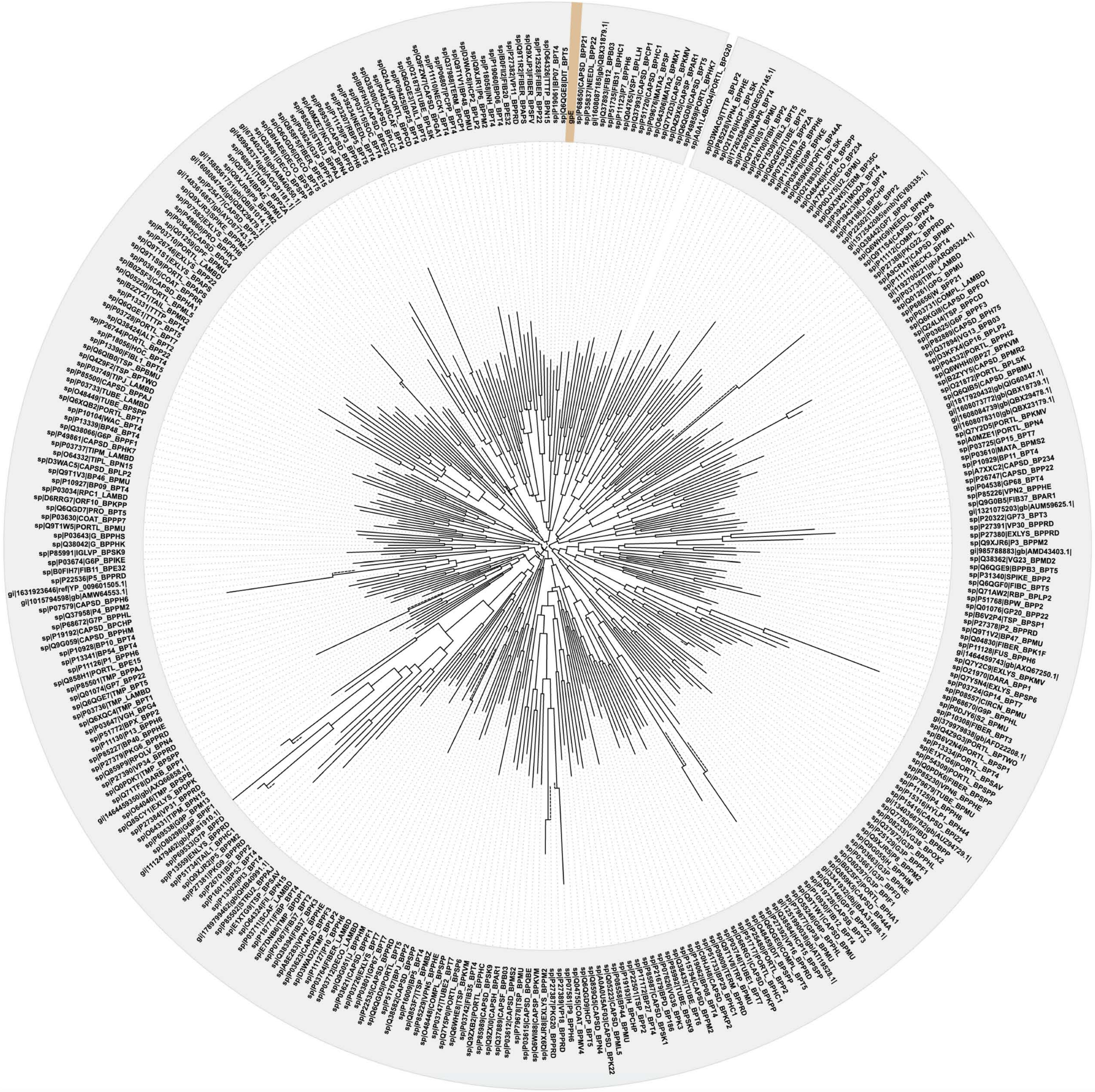

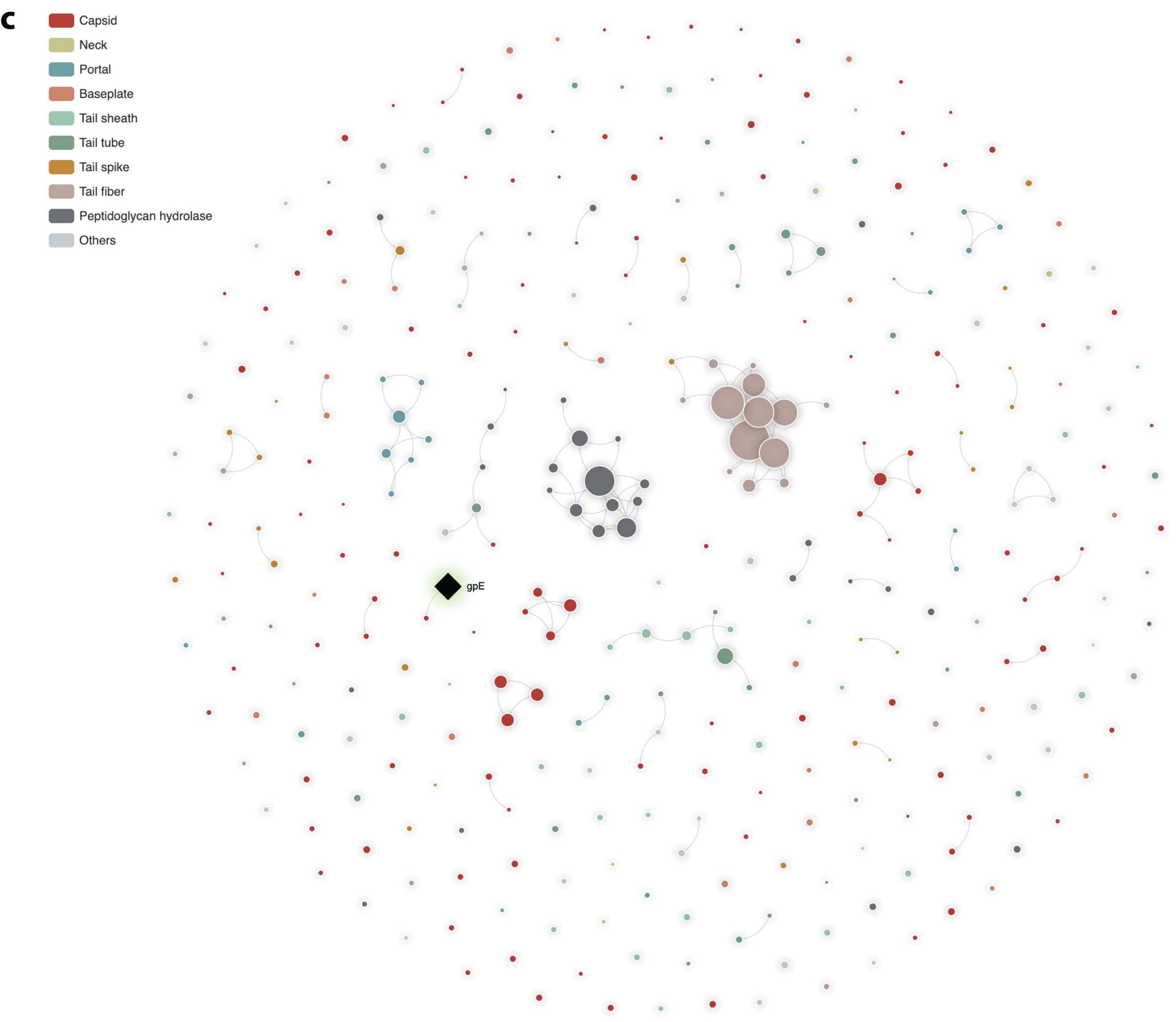
c
Capsid
Neck
Portal
Baseplate
Tail sheath
Tail tube
Tail spike
Tail fiber
Peptidoglycan hydrolase
Others
gpE

d

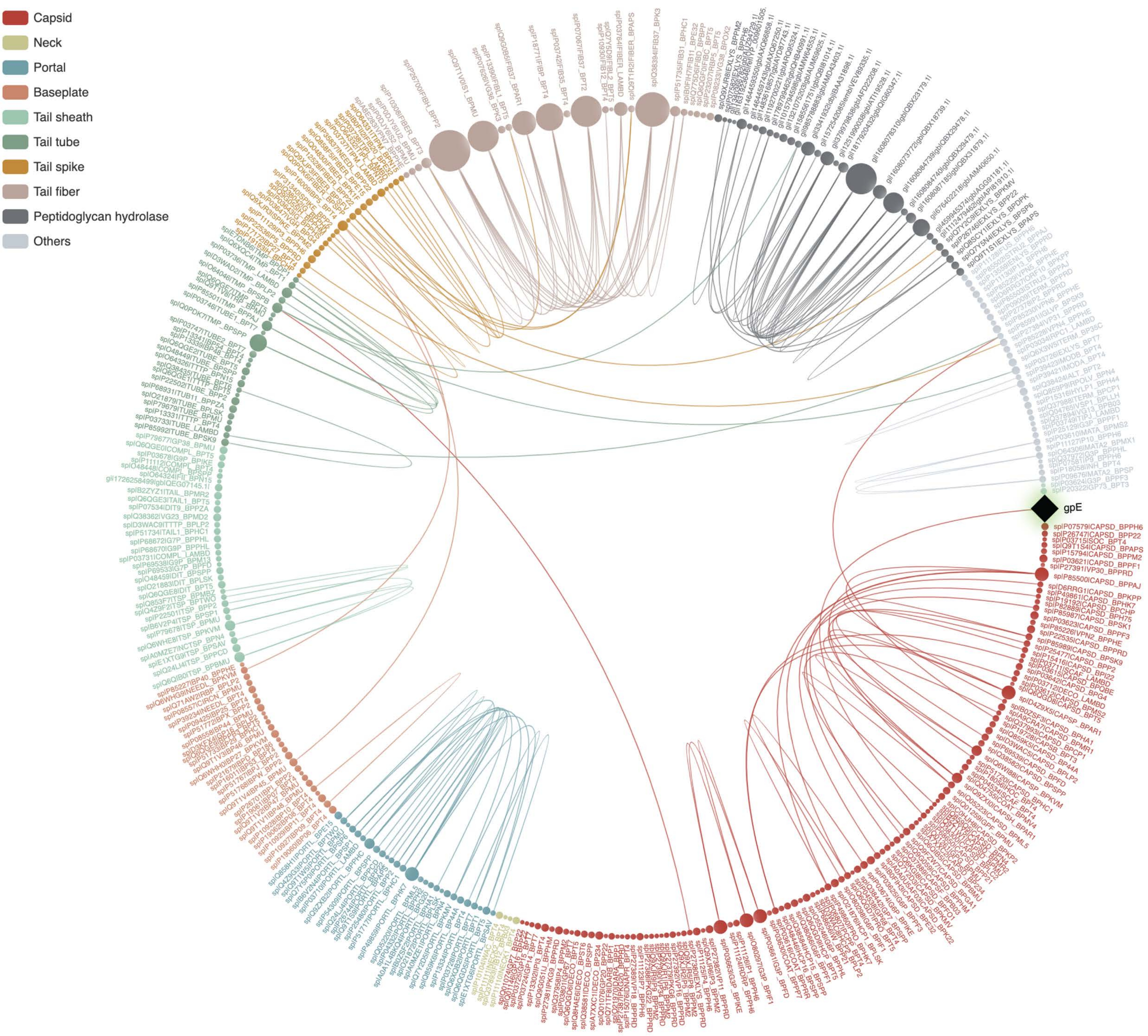

Capsid
Neck
Portal
Baseplate
Tail sheath
Tail tube
Tail spike
Tail fiber
Peptidoglycan hydrolase
Others
gpE
