# Supplementary figures and images for: Component Parts of Bacteriophage Virions Accurately Defined by a Machine-Learning Approach Built on Evolutionary Features
Source: mSystems. 2021 May 27;6(3):e00242-21. doi: 10.1128/mSystems.00242-21 (PMC8269216; doi:10.1128/mSystems.00242-21)

**a**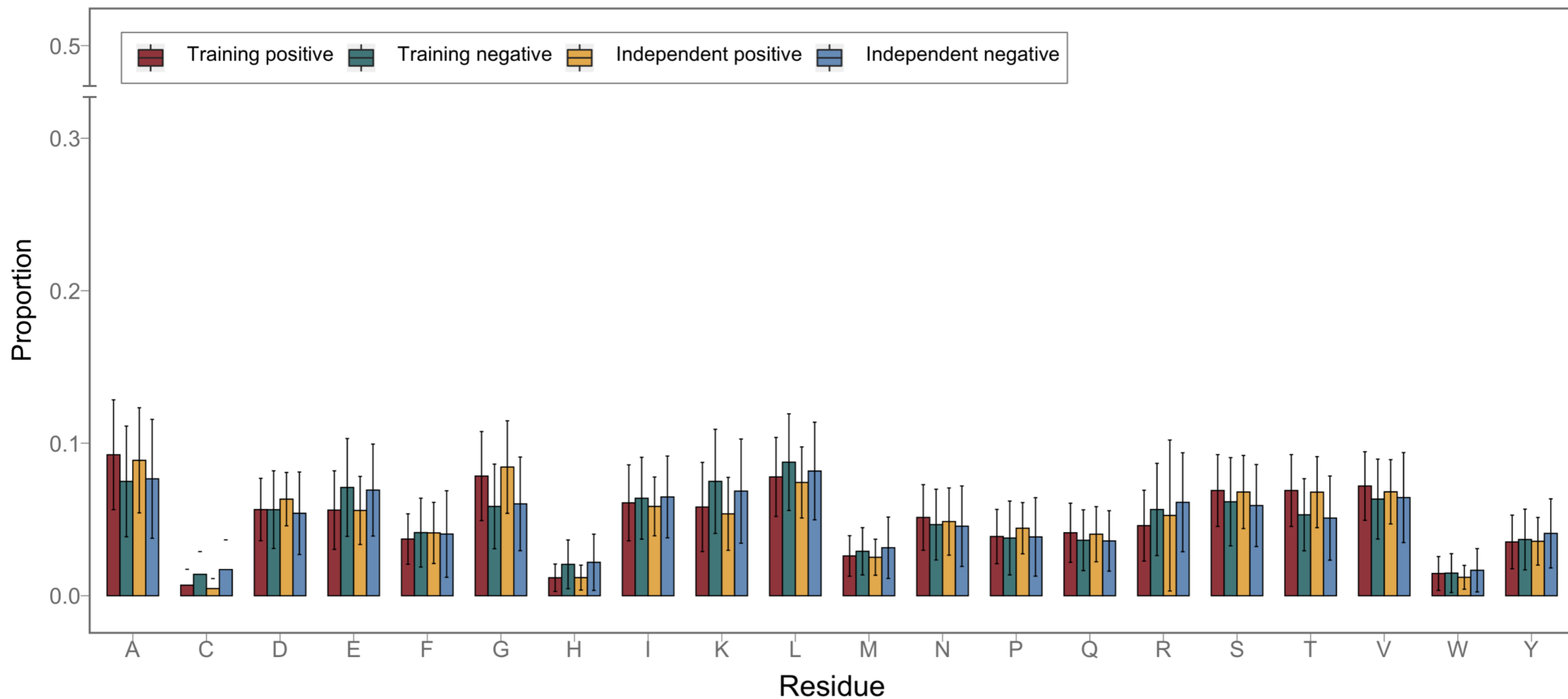**b**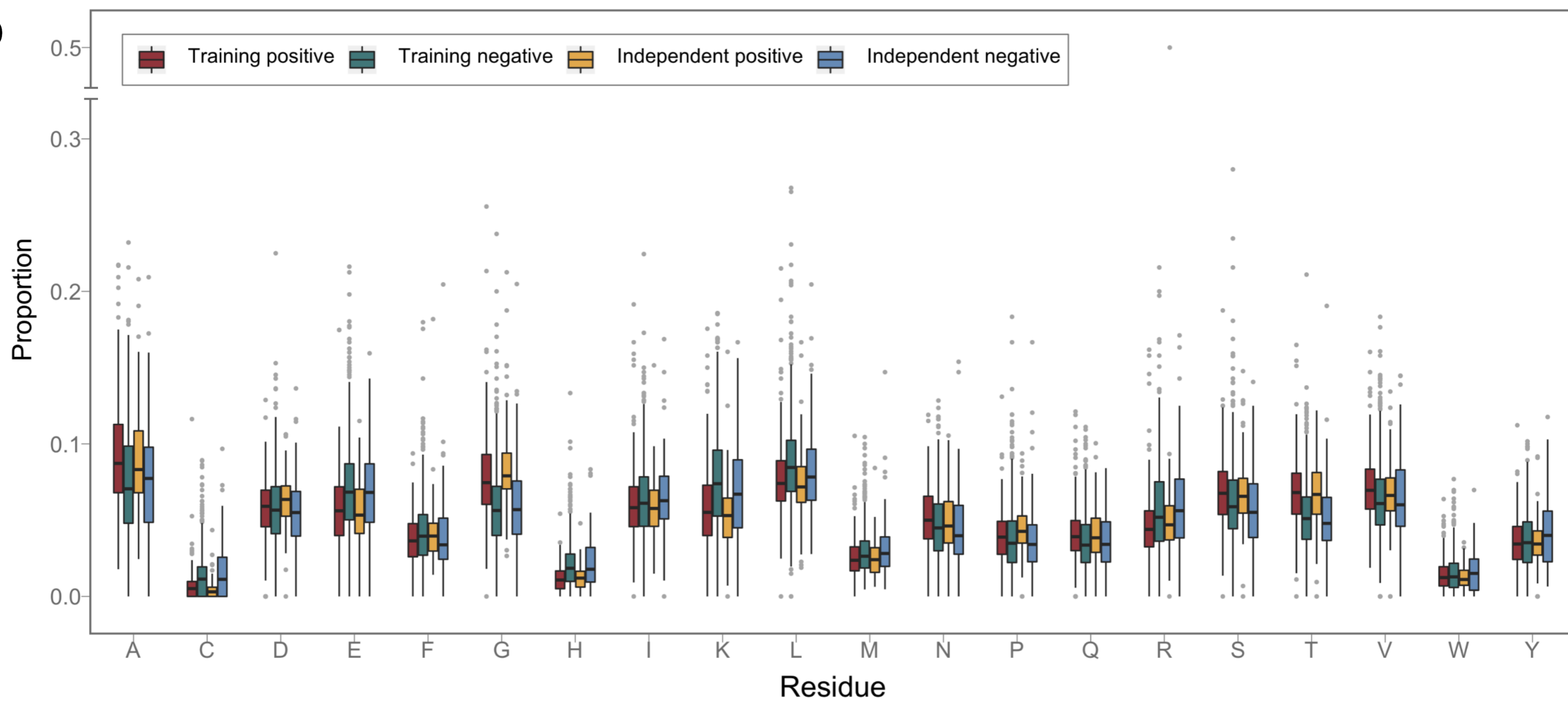

Supplement: FIG S2 [file msystems.00242-21-sf002.pdf]
