# Supplementary material for: Component Parts of Bacteriophage Virions Accurately Defined by a Machine-Learning Approach Built on Evolutionary Features
Source: mSystems. 2021 May 27;6(3):e00242-21. doi: 10.1128/mSystems.00242-21 (PMC8269216; doi:10.1128/mSystems.00242-21)
Supplement: TABLE S1 [file msystems.00242-21-st001.pdf]

| **Encoding** | **Sensitivity (SN)** | **Specificity (SP)** | **Accuracy (ACC)** | **F-value** | **Matthews correlation coefficient (MCC)** |
|---|---|---|---|---|---|
| AAC | 0.753±0.036 | 0.782±0.040 | 0.767±0.019 | 0.762±0.020 | 0.537±0.037 |
| DPC | 0.754±0.017 | 0.793±0.023 | 0.774±0.013 | 0.768±0.013 | 0.548±0.028 |
| QSOrder | 0.770±0.018 | 0.793±0.022 | 0.780±0.020 | 0.776±0.020 | 0.565±0.038 |
| PAAC | 0.760±0.019 | 0.793±0.048 | 0.778±0.023 | 0.772±0.019 | 0.558±0.043 |
| AAC-PSSM | **0.817±0.017** | 0.858±0.022 | **0.837±0.017** | **0.832±0.016** | **0.675±0.035** |
| PSSM-composition | 0.797±0.019 | 0.828±0.023 | 0.812±0.019 | 0.809±0.019 | 0.625±0.037 |
| DPC-PSSM | 0.778±0.010 | 0.844±0.016 | 0.811±0.009 | 0.803±0.009 | 0.622±0.019 |
| AADP-PSSM | 0.788±0.008 | 0.838±0.014 | 0.812±0.009 | 0.806±0.009 | 0.625±0.020 |
| MEDP | 0.807±0.025 | 0.825±0.024 | 0.816±0.020 | 0.813±0.021 | 0.631±0.041 |
| STEP[3] | 0.803±0.025 | **0.864±0.015** | 0.833±0.017 | 0.826±0.019 | 0.667±0.034 |
