# Supplementary material for: Component Parts of Bacteriophage Virions Accurately Defined by a Machine-Learning Approach Built on Evolutionary Features
Source: mSystems. 2021 May 27;6(3):e00242-21. doi: 10.1128/mSystems.00242-21 (PMC8269216; doi:10.1128/mSystems.00242-21)
Supplement: TABLE S2 [file msystems.00242-21-st002.pdf]

**Encoding**	**Sensitivity (SN)**	**Specificity (SP)**	**Accuracy (ACC)**	**F-value**	**Matthews correlation coefficient (MCC)**
AAC	0.875	0.812	0.844	0.848	0.689
DPC	0.812	0.875	0.844	0.839	0.689
QSOrder	0.844	0.844	0.844	0.844	0.688
PAAC	0.854	0.833	0.844	0.845	0.688
AAC-PSSM	0.885	0.854	0.87	0.872	0.74
PSSM-composition	**0.906**	0.844	0.875	0.879	0.751
DPC-PSSM	0.875	0.844	0.859	0.862	0.719
AADP-PSSM	0.896	0.865	0.88	0.882	0.761
MEDP	0.885	0.854	0.87	0.872	0.74
STEP[3]	0.896	**0.885**	**0.891**	**0.891**	**0.781**
