# Supplementary material for: Component Parts of Bacteriophage Virions Accurately Defined by a Machine-Learning Approach Built on Evolutionary Features
Source: mSystems. 2021 May 27;6(3):e00242-21. doi: 10.1128/mSystems.00242-21 (PMC8269216; doi:10.1128/mSystems.00242-21)
Supplement: TABLE S3 [file msystems.00242-21-st003.pdf]

**Model**	**Sensitivity (SN)**	**Specificity (SP)**	**Accuracy (ACC)**	**F-value**	**Matthews correlation coefficient (MCC)**
BLAST	0.271	**0.99**	0.63	0.423	0.375
iVIREONS	0.781	0.698	0.74	0.75	0.481
PVPred	0.385	0.875	0.63	0.51	0.299
PVP-SVM	0.427	0.865	0.646	0.547	0.324
Pred-BVP-Unb*	0.615	0.917	0.766	0.724	0.557
PVPred-SCM	0.51	0.76	0.635	0.583	0.28
$STEP^3$	**0.896**	0.885	**0.891**	**0.891**	**0.781**
