# Supplementary material for: Component Parts of Bacteriophage Virions Accurately Defined by a Machine-Learning Approach Built on Evolutionary Features
Source: mSystems. 2021 May 27;6(3):e00242-21. doi: 10.1128/mSystems.00242-21 (PMC8269216; doi:10.1128/mSystems.00242-21)
Supplement: FIG S3 [file msystems.00242-21-sf003.pdf]

a

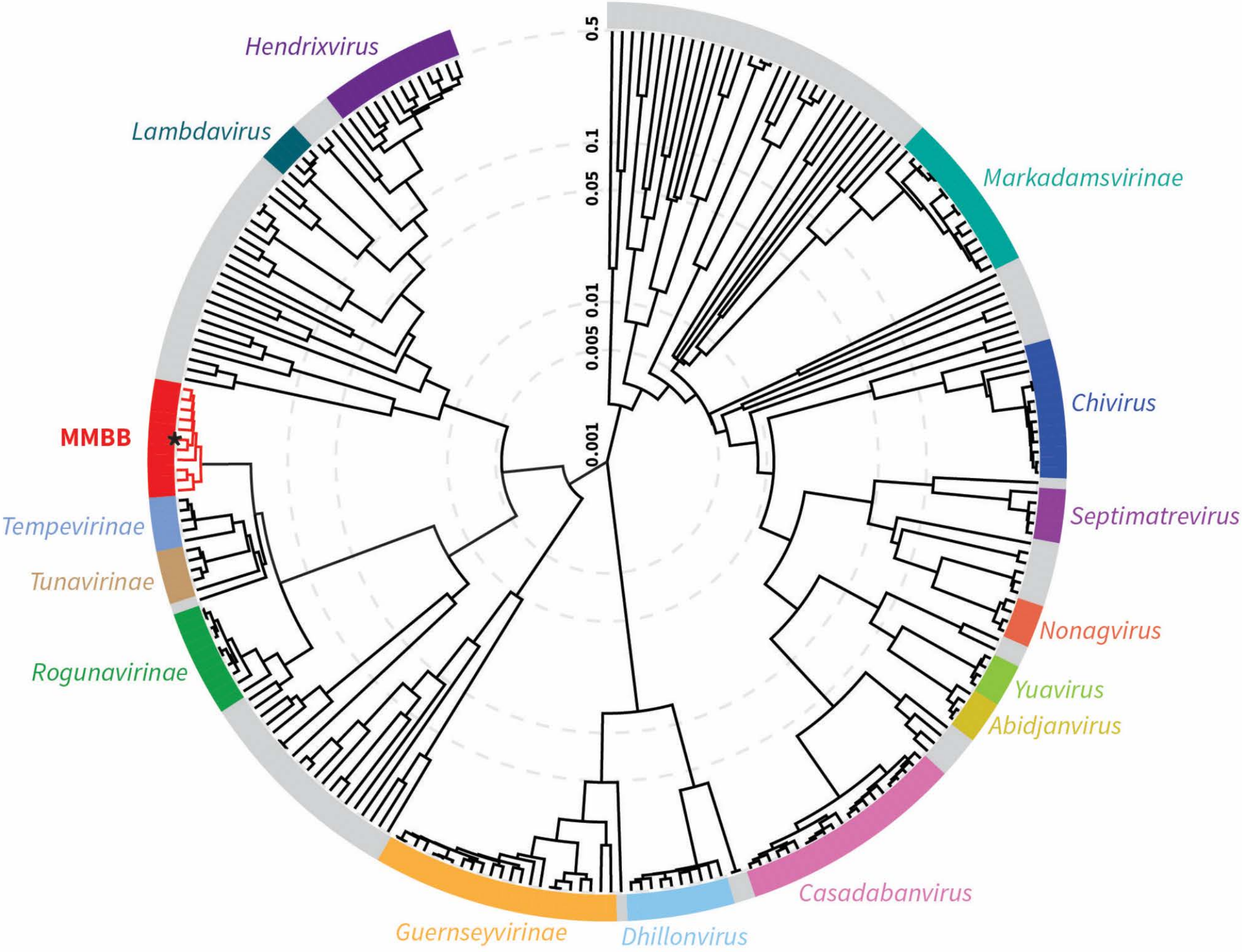

0.5
0.1
0.05
0.01
0.005
0.001
Hendrixvirus
Lambdavirus
Markadamsvirinae
Chivirus
Septimatrevirus
Nonagvirus
Yuavirus
Abidjanvirus
Casadabanvirus
Dhillonvirus
Guernseyvirinae
Rogunavirinae
Tunavirinae
Tempevirinae
MMBB

b

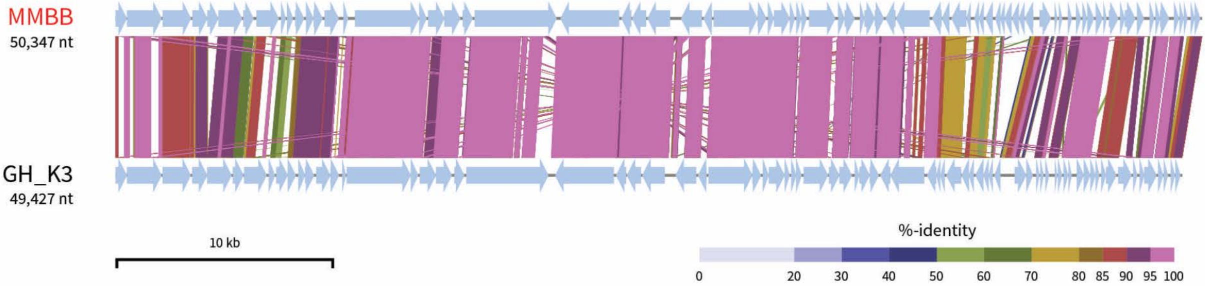

MMBB
50,347 nt
GH_K3
49,427 nt
10 kb
%-identity
0
20
30
40
50
60
70
80
85
90
95
100

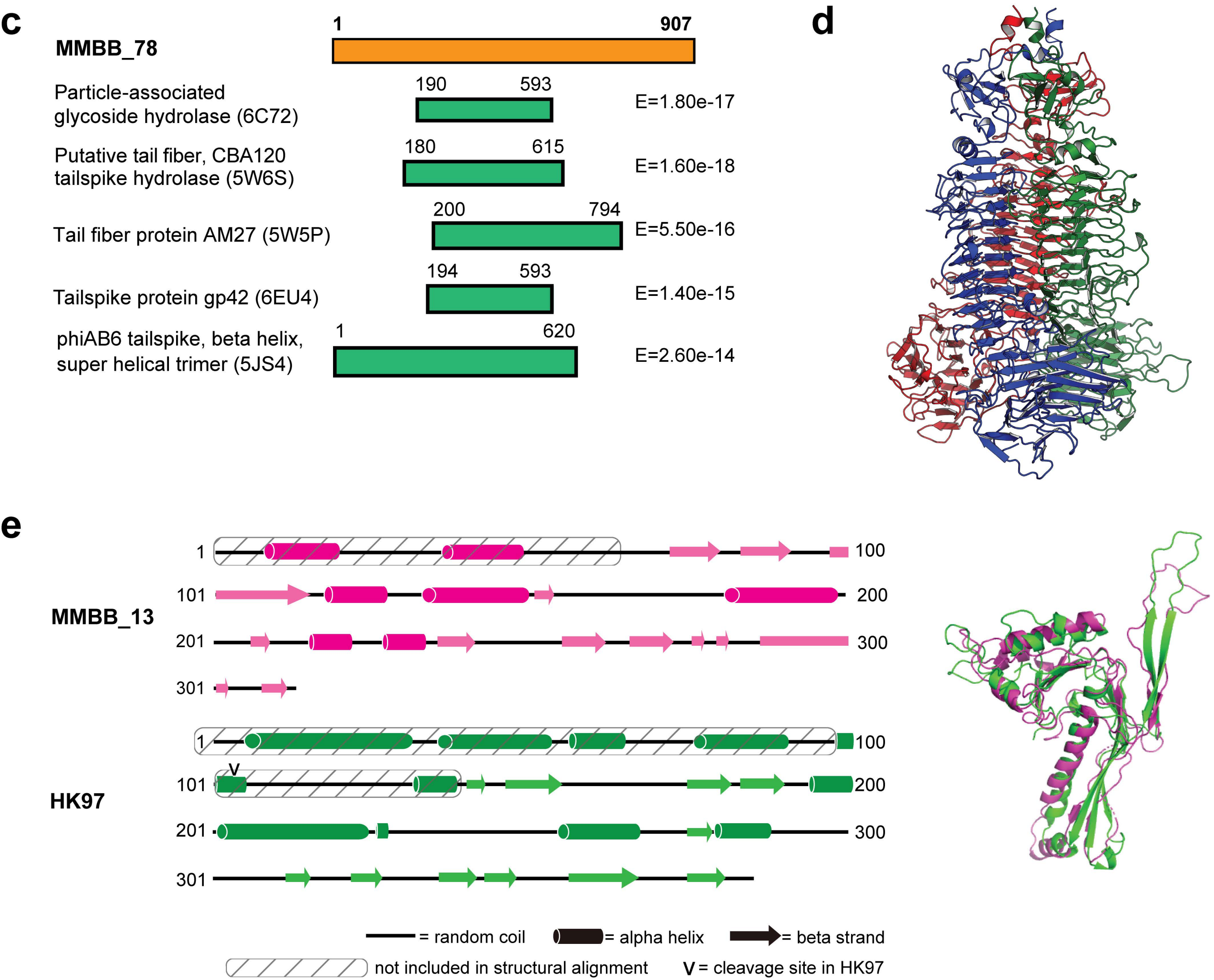
c
MMBB_78
1
907
Particle-associated glycoside hydrolase (6C72)
190
593
E=1.80e-17
Putative tail fiber, CBA120 tailspike hydrolase (5W6S)
180
615
E=1.60e-18
Tail fiber protein AM27 (5W5P)
200
794
E=5.50e-16
Tailspike protein gp42 (6EU4)
194
593
E=1.40e-15
phiAB6 tailspike, beta helix, super helical trimer (5JS4)
1
620
E=2.60e-14
d
e
MMBB_13
1
100
101
200
201
300
301
HK97
1
100
V
101
200
201
300
301
= random coil
= alpha helix
= beta strand
not included in structural alignment
V = cleavage site in HK97
