# Supplementary material for: Component Parts of Bacteriophage Virions Accurately Defined by a Machine-Learning Approach Built on Evolutionary Features
Source: mSystems. 2021 May 27;6(3):e00242-21. doi: 10.1128/mSystems.00242-21 (PMC8269216; doi:10.1128/mSystems.00242-21)
Supplement: TABLE S4 [file msystems.00242-21-st004.pdf]

**Gene**	**STEP[3]**	**Description**
MMNM_03	+	Hypothetical protein
MMNM_04	+	Hypothetical protein
MMNM_07	+	Neck protein
MMNM_08	+	Head closure protein
MMNM_09	+	Tail-completion protein
MMNM_10	+	Hypothetical protein
MMNM_11	+	Hypothetical protein
MMNM_17	+	Hypothetical protein
MMNM_18	+	Tape measure protein
MMNM_19	+	Lytic transglycosylase
MMNM_20	+	Hypothetical protein
MMNM_21	+	Baseplate protein
MMNM_22	+	Phospholipase
MMNM_23	+	Baseplate J-like protein
MMNM_24	+	Tail fibre family protein
MMNM_26	+	Base-plate wedge protein
MMNM_27	+	Putative tail-fibre protein
MMNM_47	-	HAD superfamily hydrolase
MMNM_50	-	Polynucleotide kinase (PNK)
MMNM_55	+	Portal protein
MMNM_57	-	Hypothetical protein
MMNM_58	+	Head morphogenesis protein
MMNM_65	+	Coil containing protein
MMNM_66	+	Hypothetical protein
MMNM_67	+	Major capsid protein
