# Supplementary material for: Component Parts of Bacteriophage Virions Accurately Defined by a Machine-Learning Approach Built on Evolutionary Features
Source: mSystems. 2021 May 27;6(3):e00242-21. doi: 10.1128/mSystems.00242-21 (PMC8269216; doi:10.1128/mSystems.00242-21)
Supplement: TABLE S5 [file msystems.00242-21-st005.pdf]

Gene	BLAST[a]	iVIREONS[b]	PVPred[c]	PVP-SVM[d]	Pred-BVP-Unb[e,*]	PVPred-SCM[f]	STEP[3 g]
MMNM_01	-	0.709	0.045	0.092	0.005	450.560	0.072
MMNM_02	-	-0.418	0.085	0.070	0.013	423.970	0.218
MMNM_03	-	-0.717	0.915	0.600	0.890	456.590	0.750
MMNM_04	-	0.878	0.898	0.878	0.130	537.390	0.932
MMNM_05	-	-0.961	0.010	0.005	0.024	454.120	0.116
MMNM_06	-	-0.942	0.000	0.004	0.004	441.330	0.057
MMNM_07	-	-0.497	0.573	0.684	0.404	472.100	0.689
MMNM_08	-	0.916	0.311	0.443	0.159	457.410	0.736
MMNM_09	-	0.781	0.695	0.418	0.645	457.670	0.706
MMNM_10	-	0.938	0.476	0.695	0.967	472.660	0.968
MMNM_11	-	0.995	0.563	0.711	0.918	495.130	0.873
MMNM_12	-	0.832	0.144	0.095	0.002	420.030	0.088
MMNM_13	-	-0.783	0.004	0.013	0.002	422.750	0.106
MMNM_14	-	-0.997	0.337	0.394	0.105	466.370	0.352
MMNM_15	-	0.573	0.215	0.278	0.018	429.450	0.083
MMNM_16	-	-0.645	0.630	0.516	0.261	468.390	0.466
MMNM_17	-	-0.427	0.926	0.840	0.564	473.380	0.849
MMNM_18	-	0.976	0.749	0.737	0.562	480.270	0.907
MMNM_19	√	0.988	0.345	0.475	0.968	473.730	0.868
MMNM_20	-	0.958	0.921	0.740	0.250	485.060	0.894
MMNM_21	-	0.948	0.446	0.331	0.988	464.490	0.931
MMNM_22	-	0.986	0.297	0.145	0.772	438.870	0.679
MMNM_23	√	0.996	0.838	0.556	0.997	477.930	0.952
MMNM_24	√	0.975	0.432	0.461	0.961	496.040	0.925

MMNM_25	-	-0.350	0.046	0.261	0.076	435.180	0.468
MMNM_26	-	0.996	0.741	0.888	0.803	466.610	0.802
MMNM_27	-	0.870	0.989	0.570	0.091	510.040	0.645
MMNM_28	-	-0.778	0.008	0.035	0.011	424.010	0.111
MMNM_29	-	-0.870	0.077	0.341	0.125	447.120	0.215
MMNM_30	-	0.904	0.087	0.184	0.015	417.020	0.412
MMNM_31	-	-0.134	0.068	0.145	0.148	481.890	0.385
MMNM_32	-	-0.995	0.111	0.343	0.103	488.980	0.399
MMNM_33	-	-0.353	0.486	0.215	0.058	420.260	0.205
MMNM_34	-	-0.972	0.202	0.210	0.002	454.030	0.226
MMNM_35	-	0.102	0.052	0.124	0.015	437.820	0.237
MMNM_36	-	-0.278	0.022	0.000	0.004	340.140	0.243
MMNM_37	-	-0.658	0.269	0.555	0.551	462.780	0.630
MMNM_38	-	-0.345	0.034	0.064	0.109	436.530	0.231
MMNM_39	-	-0.075	0.121	0.523	0.963	460.690	0.342
MMNM_40	-	0.662	0.157	0.278	0.027	509.800	0.238
MMNM_41	-	-0.988	0.149	0.010	0.208	367.150	0.136
MMNM_42	-	-0.443	0.087	0.136	0.128	447.920	0.269
MMNM_43	-	0.629	0.230	0.158	0.025	450.150	0.397
MMNM_44	-	-0.952	0.262	0.124	0.007	428.910	0.338
MMNM_45	-	0.456	0.063	0.066	0.003	466.480	0.142
MMNM_46	-	-0.355	0.191	0.194	0.609	469.240	0.157
MMNM_47	-	-0.769	0.021	0.032	0.024	443.840	0.136
MMNM_48	-	-0.959	0.428	0.117	0.011	495.500	0.280
MMNM_49	-	-0.924	0.111	0.070	0.630	453.810	0.199
MMNM_50	-	0.031	0.063	0.138	0.006	464.010	0.290
MMNM_51	-	-0.988	0.005	0.617	0.023	411.650	0.159

MMNM_52	-	0.523	0.007	0.019	0.004	423.960	0.101
MMNM_53	-	0.794	0.076	0.263	0.056	439.390	0.305
MMNM_54	-	-0.966	0.039	0.152	0.023	428.100	0.092
MMNM_55	-	0.874	0.165	0.459	0.284	455.270	0.929
MMNM_56	-	0.533	0.104	0.021	0.010	432.160	0.156
MMNM_57	-	-0.991	0.083	0.043	0.015	400.700	0.190
MMNM_58	-	-0.189	0.561	0.362	0.219	454.010	0.823
MMNM_59	-	-0.055	0.020	0.441	0.026	443.610	0.604
MMNM_60	-	-0.165	0.086	0.038	0.024	417.150	0.197
MMNM_61	-	0.770	0.244	0.207	0.013	468.320	0.265
MMNM_62	-	0.996	0.963	0.032	0.777	501.030	0.525
MMNM_63	-	0.919	0.392	0.279	0.142	453.180	0.600
MMNM_64	-	-0.611	0.448	0.018	0.001	454.960	0.208
MMNM_65	-	0.609	0.382	0.579	0.120	457.060	0.529
MMNM_66	-	0.199	0.917	0.935	0.856	502.110	0.948
MMNM_67	-	0.883	0.684	0.466	0.924	474.930	0.980
