# Supplementary material for: Component Parts of Bacteriophage Virions Accurately Defined by a Machine-Learning Approach Built on Evolutionary Features
Source: mSystems. 2021 May 27;6(3):e00242-21. doi: 10.1128/mSystems.00242-21 (PMC8269216; doi:10.1128/mSystems.00242-21)
Supplement: TABLE S6 [file msystems.00242-21-st006.pdf]

Gene	BLAST[a]	iVIREONS[b]	PVPred[c]	PVP-SVM[d]	Pred-BVP-Unb[e,*]	PVPred-SCM[f]	STEP[3 g]
MMBB_01	-	0.772	0.635	0.419	0.078	476.910	0.546
MMBB_02	-	-0.842	0.028	0.066	0.003	455.890	0.217
MMBB_03	√	0.926	0.370	0.198	0.278	455.820	0.793
MMBB_04	√	0.874	0.697	0.922	0.807	461.750	0.783
MMBB_05	√	0.997	0.476	0.400	0.954	465.510	0.905
MMBB_06	√	-0.845	0.167	0.367	0.021	430.440	0.521
MMBB_07	-	0.718	0.500	0.856	0.995	472.240	0.953
MMBB_08	-	-0.803	0.318	0.187	0.074	429.970	0.846
MMBB_09	-	0.852	0.747	0.545	0.773	469.600	0.897
MMBB_10	-	-0.327	0.691	0.868	0.550	486.520	0.846
MMBB_11	-	-0.425	0.190	0.209	0.077	444.040	0.668
MMBB_12	-	-0.170	0.035	0.050	0.018	392.970	0.129
MMBB_13	-	0.265	0.257	0.563	0.885	443.440	0.975
MMBB_14	-	0.963	0.272	0.429	0.793	488.430	0.931
MMBB_15	-	0.124	0.223	0.275	0.220	448.710	0.561
MMBB_16	-	-0.710	0.010	0.028	0.000	427.360	0.145
MMBB_17	-	-0.414	0.220	0.091	0.013	462.160	0.698
MMBB_18	-	0.231	0.101	0.366	0.938	443.030	0.951
MMBB_19	-	-0.652	0.087	0.218	0.007	428.060	0.110
MMBB_20	-	0.501	0.243	0.190	0.001	449.720	0.304
MMBB_21	-	-0.997	0.249	0.467	0.025	430.100	0.082
MMBB_22	-	-0.809	0.104	0.112	0.024	454.640	0.434
MMBB_23	-	-0.909	0.054	0.046	0.033	413.020	0.335
MMBB_24	-	-0.767	0.173	0.289	0.036	413.820	0.259

MMBB_25	-	0.816	0.253	0.350	0.058	454.970	0.411
MMBB_26	-	-0.872	0.006	0.100	0.001	412.840	0.110
MMBB_27	-	-0.973	0.023	0.007	0.029	396.230	0.208
MMBB_28	-	-0.629	0.067	0.074	0.165	441.720	0.245
MMBB_29	-	-0.954	0.859	0.957	0.180	506.680	0.422
MMBB_30	-	-0.955	0.029	0.252	0.103	425.270	0.217
MMBB_31	-	-0.991	0.094	0.211	0.023	451.290	0.327
MMBB_32	-	-0.984	0.008	0.103	0.018	391.850	0.137
MMBB_33	-	-0.998	0.442	0.022	0.417	431.780	0.290
MMBB_34	-	-0.343	0.322	0.542	0.026	439.710	0.243
MMBB_35	-	-0.235	0.029	0.048	0.014	434.090	0.201
MMBB_36	-	-0.196	0.251	0.140	0.031	465.340	0.439
MMBB_37	-	0.412	0.198	0.246	0.075	468.890	0.868
MMBB_38	-	-0.845	0.048	0.078	0.022	444.320	0.226
MMBB_39	-	-0.149	0.251	0.013	0.027	461.530	0.186
MMBB_40	-	-0.584	0.006	0.005	0.004	417.030	0.109
MMBB_41	-	-0.998	0.546	0.021	0.012	415.410	0.149
MMBB_42	-	-0.647	0.021	0.053	0.138	434.370	0.507
MMBB_43	-	-0.566	0.490	0.380	0.022	459.290	0.396
MMBB_44	-	-0.468	0.294	0.087	0.058	441.050	0.536
MMBB_45	-	-0.304	0.081	0.093	0.015	460.930	0.165
MMBB_46	-	-0.986	0.000	0.029	0.007	447.110	0.190
MMBB_47	-	-0.525	0.026	0.113	0.575	452.330	0.149
MMBB_48	-	-0.590	0.012	0.064	0.003	404.360	0.122
MMBB_49	-	-0.078	0.836	0.528	0.027	464.190	0.731
MMBB_50	-	-0.394	0.198	0.005	0.006	448.760	0.202
MMBB_51	-	0.981	0.883	0.720	0.050	488.660	0.550

MMBB_52	-	0.982	0.826	0.113	0.244	486.000	0.515
MMBB_53	-	-0.361	0.238	0.005	0.018	432.360	0.062
MMBB_54	-	-0.821	0.097	0.085	0.009	445.550	0.294
MMBB_55	-	-0.960	0.052	0.049	0.005	453.530	0.151
MMBB_56	-	-0.256	0.008	0.008	0.006	452.340	0.142
MMBB_57	-	-0.547	0.576	0.187	0.024	462.900	0.222
MMBB_58	-	-0.170	0.262	0.211	0.135	450.870	0.097
MMBB_59	-	0.093	0.080	0.020	0.009	428.560	0.056
MMBB_60	-	0.924	0.517	0.817	0.138	505.490	0.674
MMBB_61	-	-0.602	0.123	0.234	0.011	463.530	0.310
MMBB_62	-	0.179	0.955	0.015	0.039	487.090	0.677
MMBB_63	-	-0.998	0.031	0.082	0.105	414.570	0.359
MMBB_64	-	0.585	0.032	0.057	0.005	443.890	0.252
MMBB_65	-	-0.987	0.035	0.161	0.613	438.860	0.168
MMBB_66	-	-1.000	0.005	0.060	0.003	436.060	0.160
MMBB_67	-	-0.974	0.026	0.358	0.024	481.390	0.525
MMBB_68	-	-0.999	0.199	0.015	0.025	422.180	0.126
MMBB_69	-	-0.385	0.090	0.238	0.001	449.050	0.080
MMBB_70	-	0.173	0.019	0.119	0.028	444.000	0.352
MMBB_71	-	-0.999	0.149	0.105	0.015	452.960	0.301
MMBB_72	-	-0.411	0.140	0.113	0.106	439.260	0.272
MMBB_73	-	-0.109	0.072	0.026	0.016	446.000	0.177
MMBB_74	-	-0.852	0.015	0.136	0.110	440.660	0.250
MMBB_75	-	-0.069	0.347	0.343	0.503	447.320	0.188
MMBB_76	-	0.374	0.099	0.103	0.428	429.680	0.406
MMBB_77	-	0.992	0.225	0.110	0.007	433.260	0.415
MMBB_78	√	0.932	0.612	0.453	0.981	482.060	0.968

MMBB_79	√	0.986	0.756	0.684	0.975	475.560	0.932
