# Supplementary material for: Component Parts of Bacteriophage Virions Accurately Defined by a Machine-Learning Approach Built on Evolutionary Features
Source: mSystems. 2021 May 27;6(3):e00242-21. doi: 10.1128/mSystems.00242-21 (PMC8269216; doi:10.1128/mSystems.00242-21)
Supplement: TABLE S7 [file msystems.00242-21-st007.pdf]

Gene	STEP[3]	Description
MMBB_01	+	Putative tail assembly protein
MMBB_03	+	Putative minor tail protein
MMBB_04	+	Minor tail protein
MMBB_05	+	Tail length tape-measure protein
MMBB_06	+	Tail assembly chaperone
MMBB_07	+	Major tail protein
MMBB_08	+	Minor tail protein
MMBB_09	+	Putative neck protein
MMBB_10	+	Head-to-tail connector protein
MMBB_11	+	Head-to-tail connector complex protein
MMBB_13	+	Major capsid protein/DUF2184 protein
MMBB_14	+	Capsid decoration protein
MMBB_15	+	Major capsid protein
MMBB_16	-	AP2 domain protein
MMBB_17	+	Head morphogenesis protein
MMBB_18	+	Putative portal protein
MMBB_21	-	Hypothetical protein
MMBB_31	-	Hypothetical protein
MMBB_52	+	Hypothetical protein
MMBB_60	+	Membrane protein
MMBB_61	-	Endolysin
MMBB_62	+	Putative holin
MMBB_63	-	Nucleoside triphosphate hydrolase
MMBB_64	-	Polynucleotide kinase (PNK)
MMBB_65	-	Putative phosphoesterase
MMBB_70	-	Hypothetical protein
MMBB_76	-	Putative recombination protein
MMBB_78	+	Depolymerase
MMBB_79	+	Tail fibre protein
